# A panoramic view of RNA modifications: exploring new frontiers

**DOI:** 10.1186/s13059-018-1394-4

**Published:** 2018-01-30

**Authors:** Zijun Zhang, Eddie Park, Lan Lin, Yi Xing

**Affiliations:** 1 Bioinformatics Interdepartmental Graduate Program, University of California, Los Angeles, Los Angeles, CA 90095 USA; 2 Department of Microbiology, Immunology, & Molecular Genetics, University of California, Los Angeles, Los Angeles, CA 90095 USA

## Abstract

Meeting report on the Cold Spring Harbor Asia conference on RNA Modifications and Epitranscriptomics, held in Suzhou, China, 13–17 November, 2017.

## Introduction

Methods for transcriptome-wide analyses of RNA modifications—highlighted as the 'Method of the Year 2016' by *Nature Methods*—have recently revealed highly dynamic 'epitranscriptomes', in which RNA modifications regulate gene activities and cellular phenotypes in health and disease. The Cold Spring Harbor Asia (CSHA) conference on RNA Modifications and Epitranscriptomics highlighted cutting-edge research in this rapidly evolving field. The conference was organized by Michaela Frye (University of Cambridge, UK), Chuan He (University of Chicago, USA), Tsutomu Suzuki (University of Tokyo, Japan), and Yungui Yang (Beijing Institute of Genomics, China), and included more than 50 talks and hundreds of attendees.

## Diverse types of RNA modifications

More than 140 types of chemical modifications have been found in RNA. N^6^-methyladenosine (m^6^A) is the most prevalent type of reversible internal modification in eukaryotic mRNAs. Chuan He (University of Chicago) characterized m^6^A-modified transcripts as entering a separate track of fast decay and fast translation mediated through m^6^A reader proteins. One reader protein, YTHDF2, promotes m^6^A-mediated mRNA decay, while another reader protein, YTHDF1, promotes mRNA translation. The He group also discovered FTO and ALKBH5 as erasers of m^6^A modification, and found that these proteins have important functions. Misregulation of m^6^A-associated processes leads to stalled cell differentiation and human diseases, in particular cancer.

Another RNA modification, 5-hydroxymethylcytosine (5hmC), was characterized by Francois Fuks (Free University of Brussels (ULB), Brussels). The Fuks group developed hMeRIP-seq for transcriptome-wide mapping of 5hmC. Their studies of 5hmC characteristics in mammalian embryonic stem cells (ESCs) revealed its enrichment at the mRNA transcription start sites.

RNA tailing is another prevalent type of RNA modifications. To study the regulation of RNA tailing, Narry Kim (Seoul National University, South Korea) and colleagues developed TAIL-seq, which determines transcriptome-wide poly-A tail length and 3’ end modifications simultaneously. Using TAIL-seq, they identified CNOT as the major deadenylase and elucidated the mechanistic interactions between the two CNOT subunits, CNOT6/6L and CNOT7/8, and the poly-A binding protein PABPC1. After PABPC1 and CNOT remove poly-A tails, the uridylation-catalyzing enzymes TUT4 and TUT7 add a U-tail to deadenylated mRNAs. More recently, the Kim group discovered that guanylation is catalyzed by a subset of TUTs and slows down deadenylation. The interplays of deadenylation, uridylation, and guanylation reflect a complex regulatory network of RNA modifications at the mRNA 3’ ends.

RNA editing is an abundant RNA modification. Jian Lu (Peking University, China) discovered that adenosine-to-inosine (A-to-I) RNA editing has provided an adaptive evolutionary force through systematic analyses of RNA editing sites in *Drosophila* brains. Eddie Park (UCLA, USA) reported widespread population and allelic variation of A-to-I RNA editing in human cells and proposed a mechanism that is based on RNA secondary structure.

New types of RNA modifications are being discovered. Mark Helm (Johannes Gutenberg-University, Germany) reported an LC-MS-based method to identify novel RNA modifications. A proof-of-concept study detected KDka427, a new RNA modification with a thioacetal structure. Further applications of this method may contribute to an expanding epitranscriptome vocabulary.

## Functional impacts of RNA modifications

Elucidating the functional significance of RNA modifications has been a major goal for the field. Numerous talks focused on the impacts of RNA modifications in various biological processes and diseases.

In virology, m^6^A regulates viral infection, as shown by Stacy Horner (Duke University, USA) in *Flaviviridae*, a family of RNA viruses. Specifically, m^6^A marks the positive-sense-stranded RNA genomes of viruses in this family and regulates the viral replicative life cycle. The presence of m^6^A on viral functional elements suggests an additional layer of gene regulation in virus-host interactions.

The immune response is also regulated by RNA modifications. Brenda Bass (University of Utah, USA) presented findings on the roles of A-to-I RNA editing by ADARs (adenosine deaminases that act on RNA) and dsRNA termini-dependent cleavage by Dicer in immune response. ADARs mark cellular dsRNAs to preclude the antiviral response mediated by Dicer in invertebrates. Bass and colleagues elucidated the mechanisms of differential ATP requirement by invertebrate Dicer and its relationship with the recognition of self versus non-self dsRNAs. Xuetao Cao (Chinese Academy of Medical Sciences, China) discovered that in antiviral immune responses, DDX46 inhibits immune response by retaining m^6^A-modified antiviral transcripts in the nucleus. On the other hand, TET2 represses the expression of SOCS3, an inhibitor of the JAK3 pathway, by binding and oxidizing m^5^C in the SOCS3 3’-UTR, thus promoting infection-induced myelopoiesis. Jianlong Wang (Icahn School of Medicine at Mount Sinai, USA) also described critical roles of TET2 and RNA m^5^C oxidation in the post-transcriptional regulation of endogenous retrovirus (ERV) and ERV-associated genes in pluripotent stem cells.

Taurine modification of uridine in mitochondrial transfer RNA plays a critical role in decoding during protein synthesis. Tsutomu Suzuki (University of Tokyo, Japan) and colleagues found that the lack of this modification is a primary cause of mitochondrial diseases, and proposed 'RNA modopathy' as a new class of human diseases. Kazuhito Tomizawa (Kumamoto University, Japan) demonstrated the essential role of taurine modification and its catalyzing enzyme Mto1 in mitochondrial translation and in control of the proteostasis network, which has therapeutic implications for mitochondrial diseases.

RNA modifications have emerged as an important regulatory axis of tumorigenesis. In subtypes of acute myeloid leukemia (AML), Jianjun Chen (University of Cincinnati, USA) discovered that the m^6^A eraser FTO acts as an oncogene in certain AML cells, and that inhibiting FTO activity can suppress leukemogenesis. Furthermore, an oncometabolite, R-2-hydroxyglutarate (R-2HG), exerts a broad anti-tumor activity by inhibiting the FTO/m^6^A/MYC/CEBPA signaling axis in FTO-high AML cells.

RNA modifications play a critical role in cell fate determination. Michaela Frye (University of Cambridge, UK) demonstrated that several mechanisms of RNA modifications, in particular non-coding RNA modifications, regulate stem cell functions by providing a molecular rheostat for protein synthesis in fast response to environmental stimuli. For example, tRNA methylation responds to oxidative stress and regulates protein synthesis in stem cells.

The mRNA cap is a collection of structures on the 5’ end of mRNA that recruit proteins mediating RNA processing, export, and translation. Victoria Cowling (University of Dundee, UK) described the identification of the mRNA cap methyltransferase RAM and its regulatory role in ESC differentiation, and reported how its methyltransferase-independent characteristics had been revealed by cross-linking and immunoprecipitation sequencing (CLIP-seq) and chromatin immunoprecipitation sequencing (ChIP-seq) experiments.

Several studies highlighted the functional roles of RNA modifications in germline cells. Qi Chen (University of Nevada, USA) illustrated the intergenerational transmission of paternally acquired traits via sperm. In particular, he described paternally acquired metabolic disorders that are induced by a high-fat diet and transmitted through the epitranscriptomic markers m^5^C and m^2^G on small non-coding RNAs in mice. Mofang Liu (CAS, China) discussed the piwi/piRNA machinery in mammalian spermiogenesis and human male infertility, and revealed a novel mechanism for regulated packaging of genomic DNA into functional sperm. Ramesh Pillai (University of Geneva, Switzerland) introduced a critical role of the m^6^A reader YTHDC2 in facilitating the meiotic gene expression program during mouse spermatogenesis. In oocytes, as shown by Donal O’Carroll (MRC Center for Regenerative Medicine, UK), TUT4 and TUT7 catalyze uridylation and are required to sculpt certain transcripts for the maternal transcriptome, whereas the m^6^A reader YTHDF2 regulates transcript dosage during oocyte maturation and is a key determinant of egg quality.

Eric Phizicky (University of Rochester, USA) and colleagues characterized the highly conserved 2’-O-methyltransferase Trm7 in yeast. *Trm7* mutants grow slowly and have translation defects in *Saccharomyces cerevisiae* and *Schizosaccharomyces pombe*, and they are associated with intellectual disability in humans.

Jean-Michel Fustin (Kyoto University, Japan) presented work demonstrating the control of circadian rhythm by m^6^A modification. The mRNAs of several clock genes have high m^6^A levels, often in their 3’-UTRs, and inhibition of m^6^A stabilizes these transcripts and prolongs the circadian period.

## Interplay between RNA modifications and other RNA regulatory processes

A recurring message at the conference was the interplay between multiple types of RNA modifications and other RNA regulatory processes. For example, an important molecular consequence of RNA modifications is differential affinity for RNA-binding proteins. Yungui Yang (Beijing Institute of Genomics, China) and colleagues identified YTHDC1, an m^6^A nuclear reader that specifically recognizes m^6^A-modified transcripts and regulates exon splicing by promoting or antagonizing splicing factor binding. In a re-analysis of previously published m^6^A-LAIC-seq data and related datasets, Li Yang (CAS-MPG Partner Institute for Computational Biology, China) discovered a global downregulation of A-to-I editing in m^6^A-positive compared to m^6^A-negative RNA populations, suggesting a negative correlation between m^6^A modification and A-to-I editing.

RNA modifications also mediate mRNA translational control. Richard Gregory (Boston Children’s Hospital and Harvard University, USA) described a looping mechanism between stop-codon-associated m^6^A writer METTL3 and the eIF complex that regulates protein expression. This looping mechanism ensures that ribosomes are more efficiently recycled in METTL3-bound mRNA transcripts.

## RNA modifications in plant biology

One session of the conference was dedicated to RNA modifications in plants. Xuemei Chen (UC Riverside, USA) presented a role for the TREXII complex in miRNA biogenesis, export, and loading onto AGO1 in *Arabidopsis*. The Chen group also identified HESO1 in catalyzing pre-miRNA uridylation. Uridylation of trimmed pre-miRNAs promotes their degradation.

Rupert Fray (University of Nottingham, UK) presented a study of the m^6^A methylation complex in plants. The identified components include MTA (METTL3), MTB (METTL14), FIP37 (WTAP), Vir (KIAA1429), and Hakai, a novel m^6^A writer that may also be conserved in mammals. Guifang Jia (Peking University, China) presented the identification of the first plant m^6^A eraser, AtALKBH10B. AtALKBH10B regulates the floral transition and vegetative growth in *Arabidopsis*.

## Methods for epitranscriptome sequencing

Several speakers presented novel methods for epitranscriptome sequencing. Dan Dominissini (Tel Aviv University, Israel) presented work on Nm sequencing. Leveraging the differential reactivity of 2’-O-methylated (Nm) and 2’-hydroxylated nucleosides, Nm-seq identifies Nm sites at base resolution and characterizes the unique features of Nm modification. Chengqi Yi (Peking University, China) presented CeU-seq and m^1^A-MAP, sequencing methods for detecting pseudouridine and N^1^-methyladenosine (m^1^A), respectively. Specifically, m^1^A-MAP leverages m^1^A induced mis-incorporation to achieve single-base resolution for m^1^A site identification, revealing the enrichment of m^1^A at the 5’-cap and 5’-UTR.

Shengdong Ke (Rockefeller University, USA) presented efforts in developing m^6^A-CLIP to enable comprehensive, single-base-resolution mapping of m^6^A sites across the transcriptome. Yang Yu (CAS, China) presented a highly efficient and sensitive CLIP method called GoldCLIP that omits all gel purification steps to characterize transcriptome-wide protein-RNA interactions.

## Conclusions

Our understanding of the landscape, regulation, and functions of RNA modifications has advanced tremendously in the past few years. This CSHA conference brought together a multidisciplinary group of researchers studying RNA modifications in diverse biological systems using a broad range of approaches. With many questions still to be answered, we are excited about future scientific explorations at the new frontiers of RNA modifications and epitranscriptomics.

